# The causal relationship between serum metabolites and acne vulgaris: a Mendelian randomization study

**DOI:** 10.1038/s41598-024-61850-5

**Published:** 2024-05-14

**Authors:** Xiaoyun Wang, Yujia Wu, Pengfei Zhao, Xinren Wang, Wenjuan Wu, Jiankang Yang

**Affiliations:** 1 School of Basic Medical Sciences, Dali University, Dali, China; 2 Department of Dermatology, First Affiliated Hospital of Kunming Medical University, Kunming, China

**Keywords:** Acne vulgaris, Serum metabolites, Mendelian randomization, Causal inference, Genome-wide association, Computational biology and bioinformatics, Acne vulgaris

## Abstract

In individuals with acne vulgaris, alterations occur in serum metabolite composition, yet the exact causal link between these metabolites and acne development remains elusive. Using genome-wide association datasets, we performed bidirectional Mendelian randomization (MR) to investigate the potential causal relationship between 309 serum metabolites and acne vulgaris. We performed sensitivity analysis to evaluate the presence of heterogeneity and pleiotropy. Forward MR analysis found 14 serum metabolites significantly associated with acne vulgaris, and reverse MR analysis found no significant association between acne vulgaris and these serum metabolites. Through validation using data from the FinnGen database of acne vulgaris studies, we found a conclusive and significant correlation between stearoylcarnitine and acne vulgaris. This provides new evidence in the search for new targets for the treatment of acne vulgaris.

## Introduction

Acne vulgaris is a common chronic inflammatory skin disease with an estimated global prevalence of 9.38%^1^. Acne vulgaris usually presents as pimples, papules, pustules and nodules on the face, chest and back. Scarring, erythema and hyperpigmentation can also be observed, which can have a serious impact on the physical and mental health of patients^2–5^. Studies have shown that excessive secretion and changes in the composition of sebum, abnormal keratinization of follicular sebaceous ducts, proliferation of *Propionibacterium acnes* in hair follicles, and inflammatory reactions can lead to acne vulgaris^6^.

Serum is a light yellow, transparent liquid separated from blood after clotting and removal of fibrinogen and some clotting factors from plasma. It has the functions of immunity, maintaining acid–base balance and osmotic pressure. The composition and content of the serum changes depending on the state of the disease^7,8^. Metabolomics is a method that has emerged in recent years to study the mechanisms of disease. It is a discipline that studies the changes in metabolites after perturbation of an organism^9^. Serum metabolites are a part of metabolomics and an important body fluid sample for metabolomics^10^. Some studies have shown that the levels of some serum metabolites are altered in acne patients compared with healthy people^11,12^.

Mendelian randomization (MR) is a novel epidemiological research method based on genome-wide association studies (GWAS). It employs genetic variants, such as single nucleotide polymorphisms (SNPs), as instrumental variables to provide evidence of causality^13^. The greatest advantage of MR is that it can avoid the influence of residual confounding factors on the accuracy of association results, making the strength of association results more reliable than observational studies. In the absence of randomized controlled trials, MR is the most convincing strategy for exploring the causal relationship between exposure and outcome^14^. Compared with traditional observational studies, MR analysis can effectively avoid the interference of reverse causality and confounding factors, and thus more accurately infer the causal relationship between exposure and outcome^15,16^. MR is a method with strong scientific robustness and is increasingly popular with researchers because it is based on published GWAS data and does not require the recruitment of new patients or the design of new studies. Yi Feng et al. have used MR to investigate the causal association of blood metabolites with lung cancer, breast cancer, ovarian cancer, and glioma, and identified 132 metabolites that may have a causal role in cancer progression^17^. Other MR studies have identified risk and protective serum metabolites associated with lacunar stroke^18^. MR provides a more convenient method for investigating the factors that influence disease.

Through genetic analysis of serum metabolites, this study explored the causal relationship between serum metabolites and acne vulgaris at the genetic level, providing new evidence in the search for viable therapeutic targets for acne vulgaris.

## Materials and methods

### Study design

**Figure 1:**
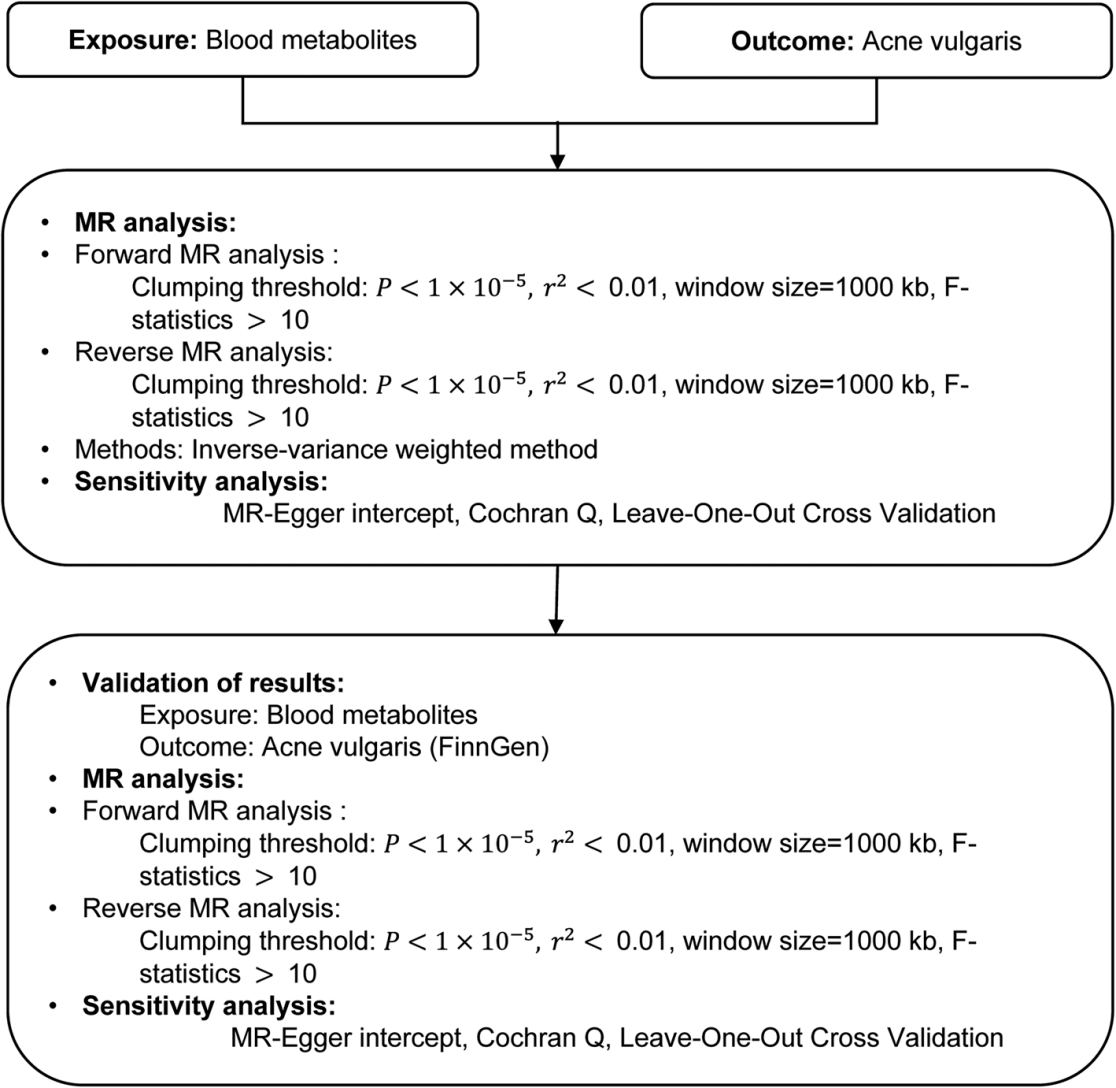
The overview of the research workflow. Abbreviations: IVW (Inverse variance weighting).

First, to identify those metabolites that might be associated with the risk of acne vulgaris, we performed a forward MR analysis using the inverse variance weighting (IVW) method. Second, to verify whether acne vulgaris would lead to changes in the screened serum metabolites, we performed a reverse MR analysis. Finally, we performed replicate analyses to exclude chance and to verify the reliability of the initial screening results. We obtained statistics on acne vulgaris from the FinnGen study and repeated the analysis using the same limiting thresholds as for the forward MR. This step allowed us to screen for serum metabolites that were ultimately associated with acne vulgaris, increasing the stability and confidence of our results (Fig. 1).

**Figure 2:**
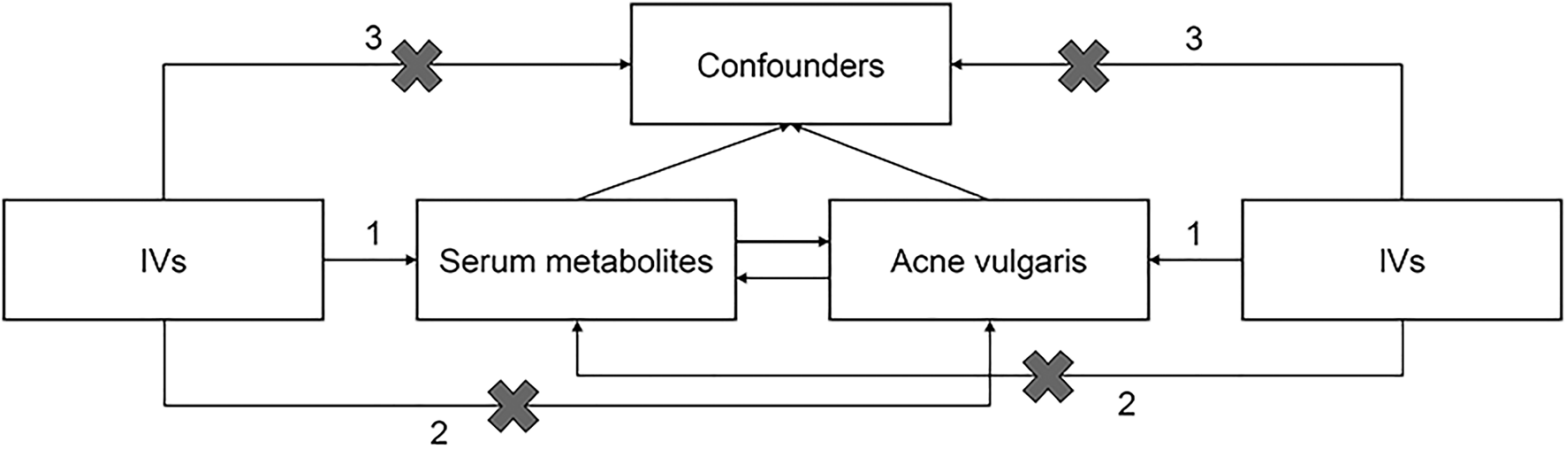
Overview of this Mendelian randomization (MR) analysis. Assumption 1: IVs must be strongly related to the exposure under study. Assumption 2: IVs cannot be related to any possible confounding factors. Assumption 3: IVs can only be related to the outcome through the exposure factor.

A valid MR study should include the following assumptions: (1) Associativity assumption: instrument variables (IVs) must be strongly related to the exposure under study, (2) Independence assumption: IVs cannot be related to any possible confounding factor, and (3) Exclusivity assumption: IVs can only be related to the outcome through the exposure factor (Fig. 2).

### Data sources

The serum metabolite data were derived from a GWAS of 486 metabolites in blood from 7824 adults from two European populations, published by SoYoun Shin et al. The 309 known metabolites were selected from this dataset^19^. The data on acne vulgaris were derived from a GWAS data of 34,422 patients with acne and 364,991 controls from three independent cohorts of European ancestry, published by Maris Teder-Laving et al. in 2023^20^.

In order to replicate the initial screening results and to rule out the possibility of chance in them, we used another acne vulgaris summary statistics from the FinnGen GWAS study (https://r10.finngen.fi/pheno/L12_ACNE). These data sources include the detailed records of 3245 patients with acne vulgaris and the corresponding information on 394,105 healthy controls^21^. The use of a large sample size enabled a more rigorous validation of the initial screening of serum metabolites associated with acne vulgaris, thereby ensuring the stability and reliability of the study results.

### Instrumental variable screening

In the forward MR analysis, when we selected a rigorous significance threshold (${P<5\times 10}^{-8}$) as the selection criterion for the IVs associated with serum metabolites but not with acne vulgaris, we could not obtain enough IVs (number of SNPs less than two). Therefore, we relaxed the significance threshold and used the following (${P<1\times 10}^{-5})$ as the selection criterion^22^. Linkage disequilibrium (LD) between SNPs was eliminated because strong LD could lead to biased results. To exclude LD interference, we used the 1000 Genomes Project European reference data, and *r*^2^ < 0.01, window size of 1000 kb was used as a screening criterion to pick out IVs^23^. All IVs were screened by calculating the F-statistic to avoid bias from weak IVs. The F-statistic of all instrumental variables should be greater than 10. Finally, 7329 SNPs were included as IVs for MR analysis.

When screening IVs for reverse MR analysis, we used the same significance thresholds and screening methods as for forward MR analysis, with ${P<1\times 10}^{-5}$, a window size of 1000 kb, and *r*^2^ < 0.01. This initiative ensured that the two methods screened the IVs with the same stringency and criteria, making the results of the two methods more comparable and consistent and enhancing the reliability and stability of the results.

### Univariate Mendelian randomization analysis of serum metabolites and acne vulgaris

In this study, IVW was used to estimate the causal effect between exposure and outcome variables. The IVW method is considered as the standard method for MR analysis, in which the causal effects of different genetic variants on traits are weighted by inverse variance. When estimating causal effects using the IVW method, the random effect model was used if there was heterogeneity, and the fixed effect model was used if there was no heterogeneity. The result was considered significant when the IVW method was statistically significant (${P}_{IVW}<0.05$)^24^. All the studies were conducted in R (version 4.3.1) using the TwoSampleMR package.

### Sensitivity analysis

To investigate the stability and reliability of the MR results, several quality control methods were used in this study. First, the Cochran Q test was used to evaluate the heterogeneity of the SNPs ($P<0.05$), indicating significant heterogeneity in the analysis results. The MR-Egger intercept test was then used to assess the presence of horizontal pleiotropy in the selected IVs when the intercept was away from the origin (${P}_{Eggger intercept}$< 0.05), indicating the potential pleiotropy of the IVs. Second, the "leave-one-out" method was used to test whether the results were robust, and the combined effect of the remaining SNPs was calculated after removing SNPs one by one to assess the effect of individual SNPs on the association between exposure and outcome variables.

### Validation of Mendelian randomization results

In MR studies, validation using different datasets is an important step in order to ensure the reliability and accuracy of the results. In order to eliminate chance and enhance the robustness of our initial screening results, we conducted a specific validation using the acne vulgaris dataset from the FinnGen study. As a large genetics research programme, the FinnGen study provides a wealth of genetic information and phenotypic data, which represents an ideal platform for validation. During the validation process, we also performed a bidirectional MR analysis. Consistent with the previous research process, we still chose a looser threshold as a screening criterion (${P<1\times 10}^{-5}$) in the validation stage. In addition, we used the same analytical methods and sensitivity tests. This not only ensures the consistency of the validation process with the previous study, but also helps us to compare the differences in results between different datasets and further assess the reliability of the results. Through this series of validation steps, we were able to be more confident that the initial screening of serum metabolites associated with acne vulgaris was authentic and reliable, providing strong evidence to support subsequent studies and the development of treatment strategies.

## Results

### Univariate Mendelian randomization analysis of serum metabolites and acne vulgaris

**Figure 3:**
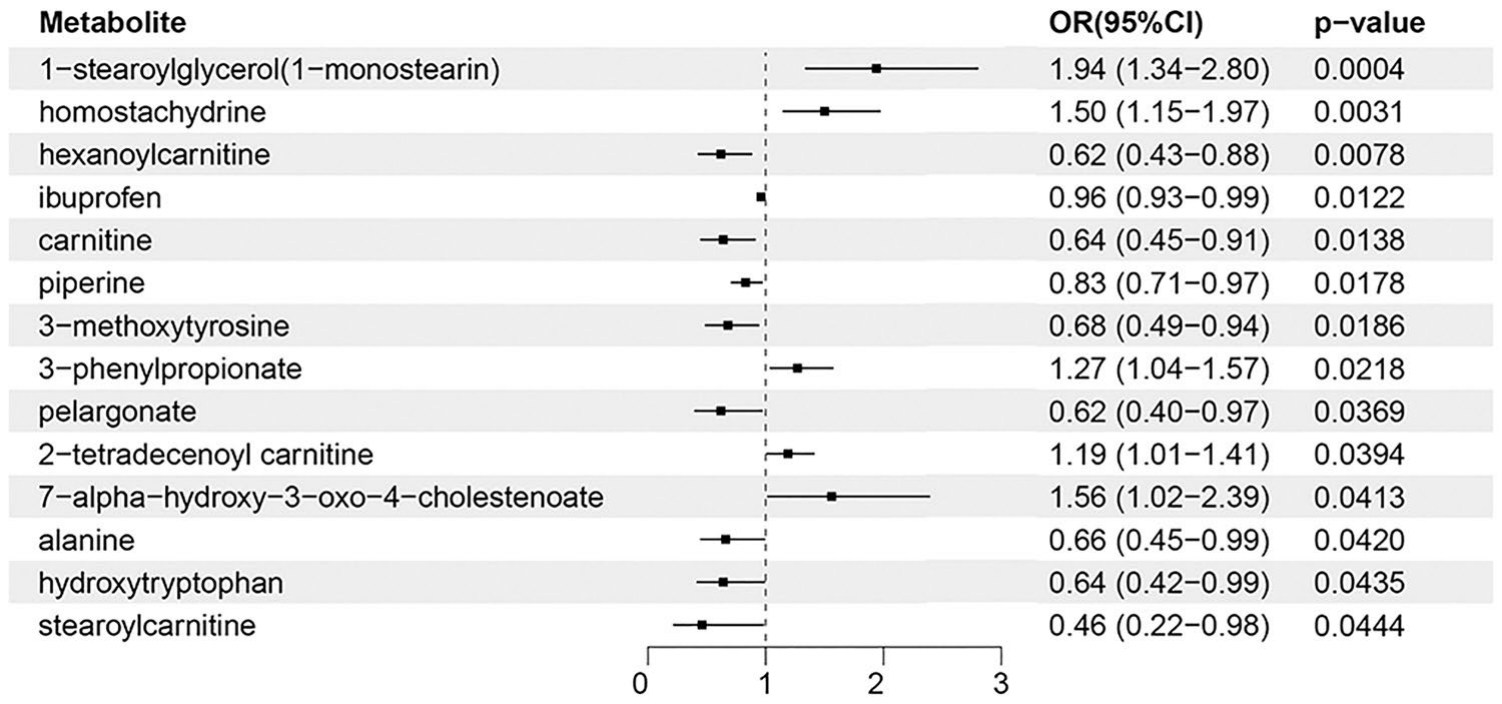
Forward MR analysis between 14 serum metabolites and acne vulgaris.

The analysis showed that the number of IVs of each metabolite was greater than 3, and the minimum F-statistic value of each IV was greater than 10, indicating adequate strength of IVs. We used the IVW method as the primary method of analysis. Given the heterogeneity of some IVs, we used the random effects model for IVW to analyze heterogeneous IVs. According to the screening criteria of our results, a total of 14 serum metabolites were confirmed to be significantly associated with acne vulgaris (Fig. 3, Table 1 and Table S1). Furthermore, leave-one-out analyses indicated that significant results were not driven by any single SNP. If all metabolites are corrected for multiple testing, none of them are significant. While correcting for multiple testing can reduce the likelihood of false positives, it can also increase the likelihood of false negatives. We will later validate the significant results without correction using another acne GWAS dataset.

**Table 1:** MR model estimated causal relationship between 14 known metabolites and the risk of acne vulgaris and tested for heterogeneity and horizontal pleiotropy. IVW, inverse variance weighting.

Exposure	Outcome	nsnp	MR analysis		Heterogeneity	Pleiotropy	OR(95%CI)
			IVW		Cochran’s Q Test (*P* Value)	Egger Intercept (*P* value)	
			beta	*P* value			
1-stearoylglycerol	Acne vulgaris	19	0.6624	0.0004	0.0885	0.8741	1.94 (1.34–2.80)
homostachydrine	Acne vulgaris	5	0.4085	0.0031	0.3344	0.169	1.50 (1.15–1.97)
hexanoylcarnitine	Acne vulgaris	15	−0.4806	0.0078	0.0535	0.4802	0.62 (0.43–0.88)
ibuprofen	Acne vulgaris	95	− 0.0391	0.0122	0.9595	0.425	0.96 (0.93–0.99)
carnitine	Acne vulgaris	177	− 0.4451	0.0138	0.0011	0.4564	0.64 (0.45–0.91)
piperine	Acne vulgaris	10	− 0.1902	0.0178	0.3361	0.6103	0.83 (0.71–0.97)
3-methoxytyrosine	Acne vulgaris	14	− 0.3911	0.0186	0.9023	0.7071	0.68 (0.49–0.94)
3-phenylpropionate	Acne vulgaris	13	0.2415	0.0218	0.9414	0.387	1.27 (1.04–1.57)
pelargonate	Acne vulgaris	31	− 0.4706	0.0369	0.0166	0.7184	0.62 (0.40–0.97)
2-tetradecenoyl carnitine	Acne vulgaris	14	0.1746	0.0394	0.549	0.5642	1.19 (1.01–1.41)
7-alpha-hydroxy-3-oxo-4-cholestenoate	Acne vulgaris	15	0.445	0.0413	0.5263	0.9912	1.56 (1.02–2.39)
alanine	Acne vulgaris	29	− 0.4086	0.042	0.414	0.06	0.66 (0.45–0.99)
hydroxytryptophan	Acne vulgaris	35	− 0.4409	0.0435	0.022	0.9304	0.64 (0.42–0.99)
stearoylcarnitine	Acne vulgaris	6	− 0.772	0.0444	0.0171	0.0736	0.46 (0.22–0.98)

### Univariate Mendelian randomization analysis of acne vulgaris and serum metabolites

**Figure 4:**
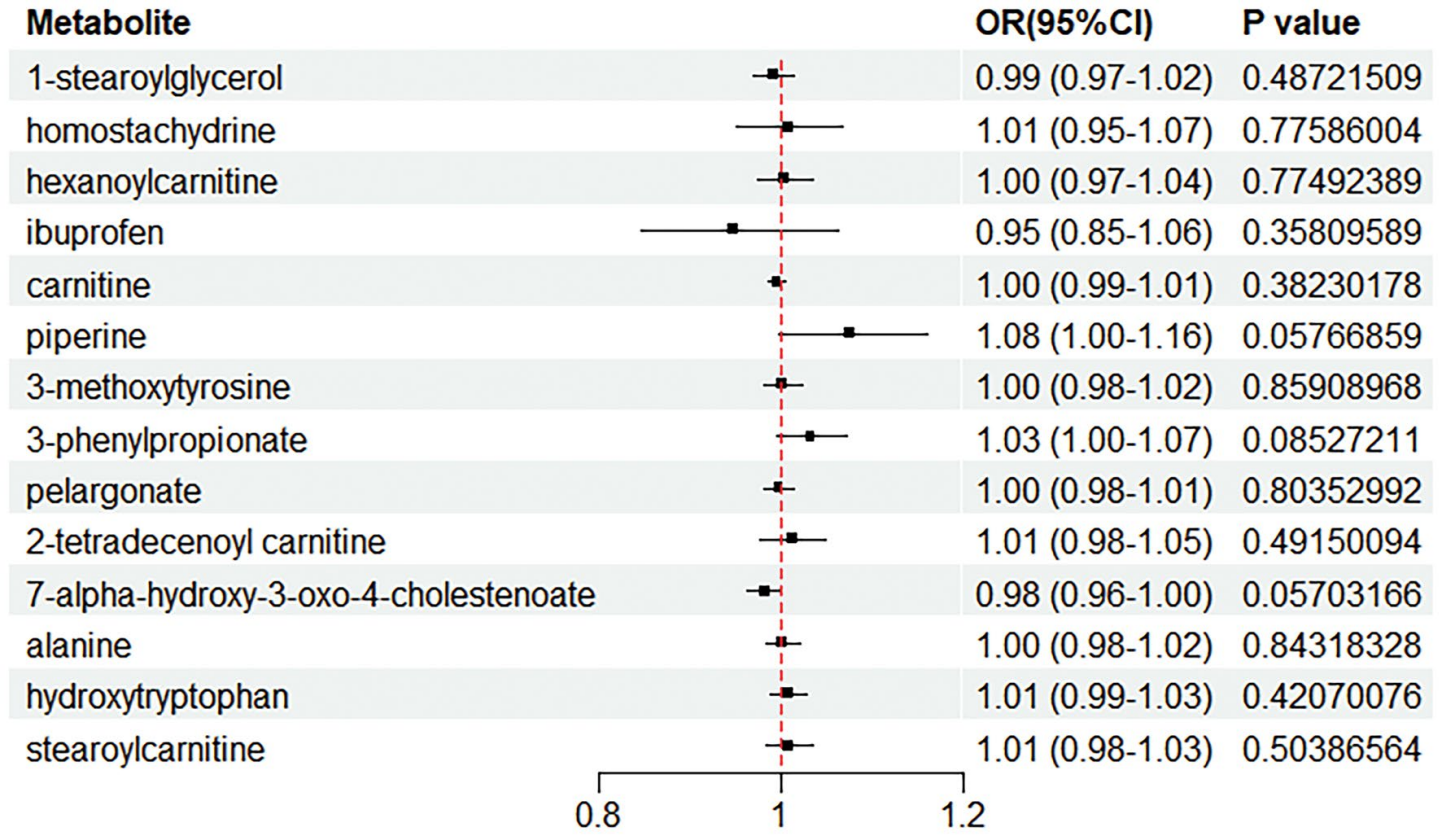
Reverse MR analysis between acne vulgaris and 14 serum metabolites.

Meanwhile, to further verify the reliability of the results of the forward MR analysis, we conducted a reverse MR analysis for the 14 significant outcomes screened above. In the reverse MR analysis, we did not find a significant causal relationship between acne vulgaris (exposure factor) and serum metabolites (outcome). This result indicated that the potential causal relationship between serum metabolites and acne vulgaris found in the forward MR analysis is unidirectional rather than bidirectional (Fig. 4 and Table 2).

**Table 2:** The inverse MR model estimated causal associations between the risk of acne vulgaris and 14 metabolites and tested for heterogeneity and horizontal pleiotropy.

Exposure	Outcome	nsnp	MR analysis		Heterogeneity	Pleiotropy	OR(95%CI)
			IVW		Cochran’s Q Test (*P* Value)	Egger Intercept (***P*** value)	
			beta	*P* value			
Acne vulgaris	1-stearoylglycerol	28	− 0.0083	0.4872	0.2256	0.2243	0.99 (0.97–1.02)
Acne vulgaris	homostachydrine	28	0.0083	0.7759	0.1424	0.5839	1.01 (0.95–1.07)
Acne vulgaris	hexanoylcarnitine	28	0.0044	0.7749	0.0531	0.8895	1.00 (0.97–1.04)
Acne vulgaris	ibuprofen	28	− 0.0535	0.3581	0.1140	0.3907	0.95 (0.85–1.06)
Acne vulgaris	carnitine	28	− 0.0044	0.3823	0.3170	0.0642	1.00 (0.99–1.01)
Acne vulgaris	piperine	28	0.0738	0.0577	0.0309	0.0407	1.08 (1.00–1.16)
Acne vulgaris	3-methoxytyrosine	28	0.0019	0.8591	0.8029	0.4738	1.00 (0.98–1.02)
Acne vulgaris	3-phenylpropionate	28	0.0325	0.0853	0.3444	0.0698	1.03 (1.00–1.07)
Acne vulgaris	pelargonate	28	− 0.0021	0.8035	0.5690	0.9093	1.00 (0.98–1.01)
Acne vulgaris	2-tetradecenoyl carnitine	28	0.0125	0.4915	0.9635	0.7515	1.01 (0.98–1.05)
Acne vulgaris	7-alpha-hydroxy-3-oxo-4-cholestenoate	28	− 0.0183	0.0570	0.9031	0.3902	0.98 (0.96–1.00)
Acne vulgaris	alanine	28	0.0020	0.8432	0.0420	0.1594	1.00 (0.98–1.02)
Acne vulgaris	hydroxytryptophan	28	0.0078	0.4207	0.1447	0.2924	1.01 (0.99–1.03)
Acne vulgaris	stearoylcarnitine	28	0.0087	0.5039	0.5681	0.4760	1.01 (0.98–1.03)

In addition, we performed a detailed heterogeneity analysis and a horizontal pleiotropy test for these results. Heterogeneity analyses were designed to assess the consistency of results across studies or datasets, while horizontal pleiotropy tests were designed to rule out situations in which genetic variation might affect multiple phenotypes simultaneously. After rigorous statistical analysis, we found no significant evidence of heterogeneity or horizontal polytropy, which further demonstrated the reliability of the results.

### Validation of Mendelian randomization results

**Figure 5:**
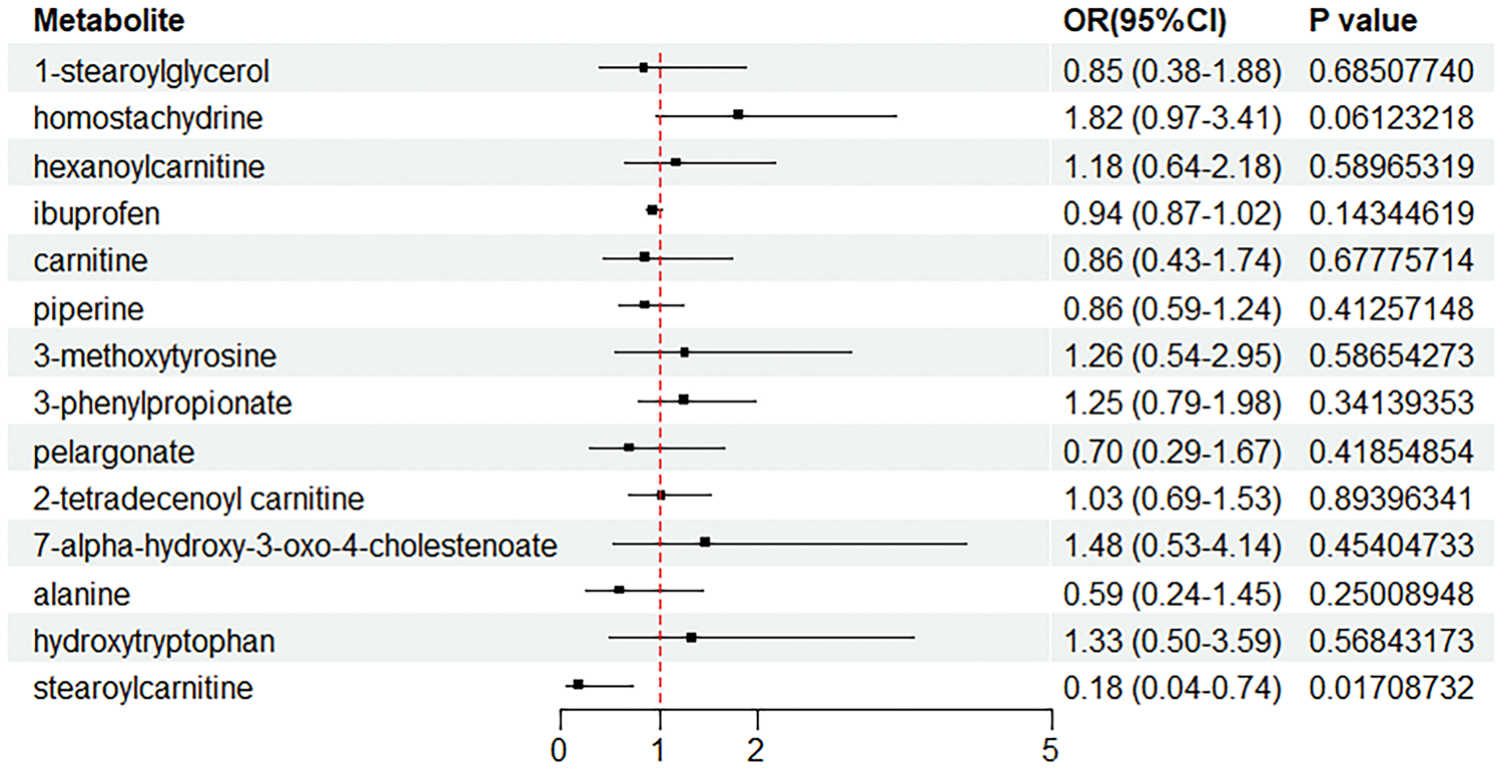
Application of forward MR analysis to explore the causal association between serum metabolites and acne vulgaris with the FinnGen database.

In order to further validate the results of the previous MR analyses on the association between serum metabolites and acne vulgaris, we conducted a validation of our results using the FinnGen database, a valuable resource that provides extensive genetic information and relevant phenotypic data, allowing us to more accurately assess the reliability of our initial screening results. We found that only stearoylcarnitine, a serum metabolite, was successfully validated, consistent with our findings in the preliminary MR analysis. This result validates that there is a potential association between stearoylcarnitine and acne vulgaris. Other results that were not successfully validated may be due to variability between different datasets, sample size limitations, or other potential confounding factors (Fig. 5 and Table 3).

**Table 3:** The forward MR validation analyses estimated causal associations between 14 known metabolites and the risk of acne vulgaris and tested for heterogeneity and horizontal pleiotropy.

Exposure	Outcome	nsnp	MR analysis		Heterogeneity	Pleiotropy	OR(95%CI)
			IVW		Cochran’s Q Test (*P* Value)	Egger Intercept (P value)	
			beta	*P* value			
1-stearoylglycerol	Acne vulgaris	24	− 0.1647	0.6851	0.3016	0.2278	0.85 (0.38–1.88)
Homostachydrine	Acne vulgaris	6	0.5996	0.0612	0.5663	0.6789	1.82 (0.97–3.41)
Hexanoylcarnitine	Acne vulgaris	18	0.1685	0.5897	0.1180	0.9955	1.18 (0.64–2.18)
Ibuprofen	Acne vulgaris	122	− 0.0573	0.1434	0.0941	0.1723	0.94 (0.87–1.02)
Carnitine	Acne vulgaris	222	− 0.1489	0.6778	0.2045	0.9634	0.86 (0.43–1.74)
Piperine	Acne vulgaris	11	− 0.1543	0.4126	0.7473	0.4871	0.86 (0.59–1.24)
3-methoxytyrosine	Acne vulgaris	16	0.2350	0.5865	0.8602	0.8764	1.26 (0.54–2.95)
3-phenylpropionate	Acne vulgaris	16	0.2231	0.3414	0.5028	0.4489	1.25 (0.79–1.98)
Pelargonate	Acne vulgaris	36	− 0.3618	0.4185	0.6410	0.4252	0.70 (0.29–1.67)
2-tetradecenoyl carnitine	Acne vulgaris	19	0.0270	0.8940	0.8962	0.2981	1.03 (0.69–1.53)
7-alpha-hydroxy-3-oxo-4-cholestenoate	Acne vulgaris	16	0.3928	0.4540	0.9401	0.8106	1.48 (0.53–4.14)
Alanine	Acne vulgaris	39	− 0.5230	0.2501	0.8606	0.1104	0.59 (0.24–1.45)
Hydroxytryptophan	Acne vulgaris	44	0.2881	0.5684	0.0517	0.7641	1.33 (0.50–3.59)
Stearoylcarnitine	Acne vulgaris	6	− 1.7073	0.0171	0.2610	0.5645	0.18 (0.04–0.74)

To further investigate the relationship between stearoylcarnitine and acne vulgaris, we performed a reverse MR analysis. Similar to the initial screening results, we did not find a significant reverse causal relationship. This result suggests that acne vulgaris itself may not directly contribute to changes in stearoylcarnitine levels (Table 4).

**Table 4:** Reverse MR validation analyses estimated the causal relationship between stearoylcarnitine and acne vulgaris and tested for heterogeneity and horizontal pleiotropy.

Exposure	Outcome	nsnp	MR analysis		Heterogeneity	Pleiotropy	OR(95%CI)
			IVW		Cochran’s Q Test (*P* Value)	Egger Intercept (*P* value)	
			beta	*P* value			
Acne vulgaris	Stearoylcarnitine	28	0.0087	0.5039	0.5681	0.4760	1.01 (0.98–1.03)

## Discussions

In this study, we performed a comprehensive MR analysis using GWAS summary statistics to assess the potential association between 309 known metabolites and acne vulgaris. Our findings revealed one statistically significant association. We eliminated the possibility of reverse causality and verified that the identified metabolites were responsible for the acne vulgaris phenotype. Stearoylcarnitine is a protective factor in acne vulgaris. Overall, our findings provide modest evidence for a causal relationship between serum metabolites and acne vulgaris.

Stearoylcarnitine is an acylcarnitine analog in human metabolism, which consists of a stearoyl group combined with a carnitine molecule. This compound may be involved in energy metabolism processes in tissues such as the heart, where stearoylcarnitine is synthesized to meet the fatty acid requirements of the tissues and maintain their normal function^25^. Stearoylcarnitine is also closely related to asthma risk. Stearoylcarnitine may affect the pathogenesis of asthma by inducing apoptosis through activation of the JNK/ERK pathway^26^.

The role of stearoylcarnitine in the pathogenesis of acne vulgaris is unknown, but may be related to cellular immunity and lipid metabolism. Although the exact mechanism of action of stearoylcarnitine in the immune system is not fully understood, studies have shown a correlation with CD4+ T cell counts and function. Firstly, stearoylcarnitine is negatively correlated with CD4+ T cell counts, which implies that elevated levels of stearoylcarnitine may lead to a decrease in the number of CD4+ T cells^27^. CD4+ T cells, as an important component of the immune system, are involved in regulating immune responses and maintaining immune homeostasis. When the number of CD4+ T cells decreases, it may affect their ability to regulate other immune cells, which in turn affects the function of the entire immune system. CD4+ T cells play a key role in the pathogenesis of acne vulgaris. They are involved in the inflammatory response in acne by producing inflammatory factors and cytokines such as IL-17 and IFN-γ. Many cytokines are regulated by the NF-κB pathway^28,29^. The release of these inflammatory factors and cytokines further exacerbates the inflammatory response of the hair follicles, leading to the formation and progression of acne lesions^30,31^.

In terms of biological activity, acylcarnitines play an important role in fatty acid metabolism, where they can participate in fatty acid transport and oxidation processes. Lipids and lipid-like substances such as stearoylcarnitine indirectly influence lysosomal function by enhancing cell membrane integrity and organelle function. Lysosomes play a role in cellular metabolism by catabolizing waste products and damaged organelles, and the action of substances such as stearoylcarnitine may lead to a limitation of lysosomal function, inhibition of the performance of the autophagy-lysosome complex, and consequently a reduction in autophagic flux^32^. In the pathogenesis of acne vulgaris, lysosomes release lysosomal substances when polymorphonuclear leukocytes come into contact with *Propionibacterium acnes*, which in turn promotes an inflammatory response in acne. Studies have shown that some substances may be present in the serum of patients with inflammatory acne that enhance the lysosomal release response and further exacerbate the inflammatory response in acne^33^. Considering the potential effect of stearoylcarnitine on lysosomal function, we can hypothesize that changes in stearoylcarnitine levels may be closely related to the inflammatory response and progression of acne vulgaris. Overall, MR is a powerful tool for studying the relationship between exposure and outcome, which can clarify causality. However, because the use of GWAS data does not allow for the investigation of potential non-linear relationships or stratification effects that vary by age, health status or sex, which may lead to heterogeneity, future MR studies or randomized controlled trials with larger sample sizes are needed to obtain more accurate results.

## Conclusions

In conclusion, our preliminary MR results identified 14 serum metabolites associated with acne vulgaris, and a causal association between stearoylcarnitine and acne vulgaris was determined after validation with data from the FinnGen study. These serum metabolites provide a new reference direction for the treatment of acne vulgaris.

### Supplementary Information

Supplementary Table 1.

## Data Availability

GWAS data of Acne vulgaris can be found here: https://www.ebi.ac.uk/gwas/publications/36922633. GWAS data of metabolomics can be found here: http://metabolomics.helmholtz-muenchen.de/gwas. GWAS data of Acne vulgaris in FinnGen can be found here: https://r10.finngen.fi/pheno/L12_ACNE.
