# Supplementary material for: The causal relationship between serum metabolites and acne vulgaris: a Mendelian randomization study
Source: Sci Rep. 2024 May 14;14:11045. doi: 10.1038/s41598-024-61850-5 (PMC11093973; doi:10.1038/s41598-024-61850-5)
Supplement: Supplementary file 1 — Supplementary Table 1. [file 41598_2024_61850_MOESM1_ESM.pdf]

The causal relationship between serum metabolites and acne vulgaris: a Mendelian randomized study

Xiaoyun Wang[1,†], Yujia Wu[1,†], Pengfei Zhao[1], Xinren Wang[1], Wenjuan Wu[2,*] and Jiankang Yang[1,*]

1 Supplementary Figures and Tables

1.1 Supplementary tables

Supplementary Table1. Two MR Models estimated the causal relationship between 309 known metabolites and the risk of acne vulgaris and tested for heterogeneity and horizontal pleiotropy.

Outcome	Exposure	nsnp	MR analysis				Heterogeneity	Pleiotropy	OR(95%CI)
			Inverse Variance Weighted		Weighted Median		Cochran's Q Test (P-Value)	Egger Intercept (P-value)	
			beta	P-value	beta	P-value			
Acne Vulgaris	1-stearoylglycerol (1-monostearin)	19	0.6624	0.0004	0.5404	0.0178	0.088488	0.8741	1.939[1.571, 2.307]
Acne	homostachydrine*	5	0.4085	0.0031	0.2773	0.1256	0.334417	0.1690	1.505[1.234,

Vulgaris									1.775]
Acne Vulgaris	hexanoylcarnitine	15	-0.4806	0.0078	-0.2703	0.2244	0.053451	0.4802	0.618[0.264, 0.972]
Acne Vulgaris	ibuprofen	95	-0.0391	0.0122	-0.0329	0.1676	0.959472	0.4250	0.962[0.931, 0.992]
Acne Vulgaris	carnitine	177	-0.4451	0.0138	-0.7052	0.0034	0.001077	0.4564	0.641[0.286, 0.995]
Acne Vulgaris	piperine	10	-0.1902	0.0178	-0.1615	0.1244	0.336135	0.6103	0.827[0.669, 0.984]
Acne Vulgaris	3-methoxytyrosine	14	-0.3911	0.0186	-0.3008	0.1855	0.902289	0.7071	0.676[0.350, 1.002]
Acne	3-phenylpropionate	13	0.2415	0.0218	0.2891	0.0434	0.941409	0.3870	1.273[1.067,

Vulgaris	(hydrocinnamate)								1.480]
Acne Vulgaris	3-dehydrocarnitine*	23	-0.3704	0.0253	-0.1723	0.4730	0.251905	0.0301	0.690[0.366, 1.015]
Acne Vulgaris	mannose	16	0.3450	0.0280	0.6135	0.0021	0.270704	0.0158	1.412[1.104, 1.720]
Acne Vulgaris	pelargonate (9:0)	31	-0.4706	0.0369	-0.4828	0.0537	0.016617	0.7184	0.625[0.183, 1.067]
Acne Vulgaris	2-tetradecenoyl carnitine	14	0.1746	0.0394	0.1797	0.1149	0.549014	0.5642	1.191[1.025, 1.357]
Acne Vulgaris	7-alpha-hydroxy-3-oxo-4-cholestenoate (7-Hoca)	15	0.4450	0.0413	0.4137	0.1543	0.526334	0.9912	1.560[1.133, 1.988]
Acne	alanine	29	-0.4086	0.0420	-0.3809	0.1948	0.413996	0.0600	0.665[0.271,

Vulgaris									1.058]
Acne Vulgaris	X-12100--hydroxytryptophan*	35	-0.4409	0.0435	-0.3371	0.2029	0.021996	0.9304	0.643[0.215, 1.071]
Acne Vulgaris	stearoylcarnitine	6	-0.7720	0.0444	-0.5012	0.1943	0.017119	0.0736	0.462[-0.291, 1.215]
Acne Vulgaris	1-methylurate	15	-0.1513	0.0505	-0.2183	0.0306	0.655444	0.5115	0.860[0.708, 1.011]
Acne Vulgaris	pentadecanoate (15:0)	14	0.3909	0.0544	0.3876	0.1774	0.761148	0.8128	1.478[1.080, 1.877]
Acne Vulgaris	pipecolate	14	-0.2264	0.0661	-0.3207	0.0515	0.939139	0.9249	0.797[0.556, 1.039]
Acne	proline	30	0.2723	0.0732	0.2415	0.2973	0.633246	0.2944	1.313[1.015,

Vulgaris									1.611]
Acne Vulgaris	mannitol	12	-0.0914	0.0798	-0.1111	0.1154	0.825040	0.5387	0.913[0.810, 1.015]
Acne Vulgaris	ADSGEGDFXAEGGGVR*	10	-0.1466	0.0800	-0.1582	0.1311	0.386497	0.6515	0.864[0.700, 1.028]
Acne Vulgaris	asparagine	30	0.2703	0.0822	0.0984	0.6755	0.730534	0.5990	1.310[1.006, 1.615]
Acne Vulgaris	X-12095--N1-methyl-3-pyridone-4-carboxamide	22	-0.2034	0.0845	-0.1890	0.2512	0.414441	0.5338	0.816[0.585, 1.047]
Acne Vulgaris	linoleate (18:2n6)	10	0.5780	0.0864	0.1069	0.7927	0.186717	0.3709	1.782[1.122, 2.443]
Acne	butyrylcarnitine	1005	-0.0295	0.0914	-0.0182	0.6654	0.033561	0.8993	0.971[0.937,

Vulgaris									1.005]
Acne Vulgaris	N-(2-furoyl)glycine	19	-0.0306	0.1021	-0.0294	0.2666	0.383360	0.6139	0.970[0.933, 1.007]
Acne Vulgaris	cholesterol	32	-0.3626	0.1030	-0.3033	0.3396	0.358160	0.6294	0.696[0.260, 1.132]
Acne Vulgaris	3-methylhistidine	9	0.1158	0.1091	0.1353	0.1697	0.662421	0.1587	1.123[0.981, 1.265]
Acne Vulgaris	caffeine	11	-0.0840	0.1109	-0.0522	0.4601	0.836908	0.2819	0.919[0.816, 1.023]
Acne Vulgaris	1-palmitoylglycerol (1-monopalmitin)	12	-0.3705	0.1130	-0.1306	0.6769	0.316240	0.6081	0.690[0.232, 1.149]
Acne	7-methylguanine	10	0.2320	0.1220	0.3273	0.1147	0.403073	0.3156	1.261[0.967,

Vulgaris									1.555]
Acne Vulgaris	1-eicosatrienoylglycerophosphocholine*	17	-0.1914	0.1291	-0.1544	0.3783	0.488235	0.9202	0.826[0.579, 1.073]
Acne Vulgaris	threonine	18	0.3864	0.1315	0.2654	0.3622	0.055643	0.7357	1.472[0.970, 1.974]
Acne Vulgaris	4-ethylphenylsulfate	6	-0.1540	0.1331	-0.2052	0.1195	0.419274	0.8618	0.857[0.656, 1.058]
Acne Vulgaris	propionylcarnitine	25	-0.2342	0.1346	-0.3060	0.1902	0.879824	0.9235	0.791[0.484, 1.098]
Acne Vulgaris	X-14977--vanillin	5	-0.2964	0.1358	-0.2950	0.2562	0.652008	0.8858	0.744[0.354, 1.133]
Acne	2-hydroxypalmitate	39	-0.2295	0.1392	-0.2930	0.2179	0.748262	0.6765	0.795[0.491,

Vulgaris									1.099]
Acne Vulgaris	#N/A	27	0.2562	0.1413	-0.0918	0.6926	0.160087	0.0174	1.292[0.951, 1.633]
Acne Vulgaris	bilirubin (E,Z or Z,E)*	14	0.1938	0.1414	0.3161	0.0223	0.014621	0.0268	1.214[0.956, 1.472]
Acne Vulgaris	pseudouridine	24	-0.3958	0.1427	-0.5399	0.1655	0.327985	0.4419	0.673[0.144, 1.202]
Acne Vulgaris	homocitrulline	6	-0.2452	0.1438	-0.3025	0.1687	0.711537	0.7314	0.783[0.454, 1.111]
Acne Vulgaris	chiro-inositol	12	0.0414	0.1527	0.0307	0.4199	0.702356	0.4672	1.042[0.986, 1.099]
Acne	kynurenine	34	-0.2676	0.1536	-0.3269	0.1986	0.086251	0.1152	0.765[0.398,

Vulgaris									1.133]
Acne Vulgaris	1-palmitoylglycerophosphocholine	24	0.3388	0.1572	0.1923	0.5917	0.650697	0.1305	1.403[0.934, 1.873]
Acne Vulgaris	pyroglutamylglycine	4	0.2075	0.1603	0.0449	0.7591	0.130405	0.9568	1.231[0.941, 1.520]
Acne Vulgaris	octadecanedioate	8	0.2198	0.1628	0.1556	0.4502	0.540624	0.7342	1.246[0.937, 1.554]
Acne Vulgaris	X-14304--leucylalanine	18	0.0937	0.1680	0.0653	0.4965	0.662976	0.2061	1.098[0.965, 1.232]
Acne Vulgaris	myristate (14:0)	19	-0.2214	0.1685	-0.4014	0.0605	0.221508	0.0967	0.801[0.486, 1.117]
Acne	gamma-glutamylglut	15	0.2931	0.1704	0.2621	0.3783	0.324517	0.4405	1.341[0.921,

Vulgaris	amine								1.760]
Acne Vulgaris	deoxycholate	15	0.0900	0.1709	0.1073	0.2255	0.888085	0.5038	1.094[0.965, 1.223]
Acne Vulgaris	fructose	3	0.4415	0.1729	0.4203	0.2621	0.969745	0.8527	1.555[0.920, 2.190]
Acne Vulgaris	dihomo-linoleate (20:2n6)	9	0.3664	0.1745	0.3050	0.2903	0.026536	0.6247	1.443[0.914, 1.971]
Acne Vulgaris	2-oleoylglycerophosphocholine*	10	-0.3514	0.1761	-0.2858	0.3863	0.308935	0.5264	0.704[0.194, 1.213]
Acne Vulgaris	p-cresol sulfate	11	-0.1117	0.1847	-0.1269	0.2699	0.821306	0.3598	0.894[0.729, 1.059]
Acne	bilirubin (Z,Z)	9	-0.1052	0.1867	-0.0580	0.5811	0.723919	0.8578	0.900[0.744,

Vulgaris								1.056]
Acne Vulgaris	1-stearoylglycerophosphoethanolamine	13	-0.2327	0.1986	-0.2350	0.1724	0.010931	0.3694	0.792[0.438, 1.147]
Acne Vulgaris	1-arachidonoylglycerophosphoethanolamine*	20	0.2421	0.2010	0.3201	0.2189	0.151791	0.4891	1.274[0.903, 1.645]
Acne Vulgaris	malate	13	0.3444	0.2042	0.3453	0.2244	0.048815	0.3673	1.411[0.879, 1.943]
Acne Vulgaris	gamma-glutamylglutamate	10	0.1015	0.2045	0.0870	0.4152	0.871672	0.3932	1.107[0.950, 1.264]
Acne Vulgaris	bradykinin, des-arg(9)	15	-0.0533	0.2093	-0.0713	0.1846	0.096663	0.8322	0.948[0.865, 1.031]
Acne	citrulline	38	-0.2332	0.2108	0.0445	0.8684	0.352705	0.9851	0.792[0.427,

Vulgaris									1.157]
Acne Vulgaris	glycocholate	7	0.1227	0.2142	0.0882	0.5137	0.688798	0.7750	1.131[0.937, 1.324]
Acne Vulgaris	cyclo(leu-pro)	14	0.1063	0.2214	0.0618	0.5745	0.165871	0.3222	1.112[0.942, 1.283]
Acne Vulgaris	androsterone sulfate	17	0.0532	0.2266	0.0444	0.3257	0.231422	0.4192	1.055[0.968, 1.141]
Acne Vulgaris	laurylcarnitine	13	-0.1909	0.2274	-0.2767	0.0783	0.012247	0.6562	0.826[0.516, 1.136]
Acne Vulgaris	1-methylxanthine	11	-0.1780	0.2277	-0.1443	0.2354	0.015939	0.6333	0.837[0.548, 1.126]
Acne	7-methylxanthine	8	-0.1176	0.2312	-0.0543	0.6565	0.895477	0.4705	0.889[0.697,

Vulgaris									1.082]
Acne Vulgaris	citrate	34	0.2202	0.2329	0.5096	0.0595	0.419056	0.0170	1.246[0.885, 1.608]
Acne Vulgaris	3-hydroxybutyrate (BHBA)	8	0.0678	0.2338	0.0928	0.1945	0.658802	0.3644	1.070[0.959, 1.182]
Acne Vulgaris	caproate (6:0)	34	-0.2717	0.2397	-0.3012	0.3043	0.010909	0.1912	0.762[0.309, 1.215]
Acne Vulgaris	arachidonate (20:4n6)	17	0.2722	0.2464	0.1773	0.5779	0.206458	0.0909	1.313[0.853, 1.773]
Acne Vulgaris	X-11423--O-sulfo-L-tyrosine	39	-0.2444	0.2521	-0.2872	0.3202	0.060672	0.7153	0.783[0.365, 1.201]
Acne	threonate	12	-0.1654	0.2532	-0.1070	0.5836	0.269339	0.2165	0.848[0.564,

Vulgaris									1.131]
Acne Vulgaris	tyrosine	23	0.2963	0.2540	0.2056	0.5666	0.351658	0.9300	1.345[0.836, 1.854]
Acne Vulgaris	salicylate	14	0.0173	0.2588	0.0159	0.4346	0.351574	0.5892	1.017[0.987, 1.047]
Acne Vulgaris	X-12244--N-acetylcarnosine	18	-0.1915	0.2638	-0.3198	0.1754	0.548761	0.0683	0.826[0.490, 1.162]
Acne Vulgaris	creatinine	22	-0.3434	0.2671	-0.3433	0.4194	0.461848	0.6867	0.709[0.103, 1.316]
Acne Vulgaris	trans-4-hydroxyproline	6	0.1602	0.2706	0.0507	0.7929	0.744808	0.6229	1.174[0.889, 1.459]
Acne	urea	10	-0.3180	0.2751	-0.4711	0.2114	0.312424	0.8778	0.728[0.157,

Vulgaris									1.299]
Acne Vulgaris	1-oleoylglycerophosphoethanolamine	8	-0.3802	0.2760	-0.0642	0.8410	0.006133	0.4279	0.684[0.000, 1.368]
Acne Vulgaris	1-palmitoylglycerophosphoinositol*	8	0.1525	0.2778	0.1936	0.3147	0.559893	0.3164	1.165[0.889, 1.440]
Acne Vulgaris	2-hydroxyhippurate (salicylurate)	11	0.0228	0.2803	0.0020	0.9446	0.841329	0.3037	1.023[0.982, 1.064]
Acne Vulgaris	decanoylcarnitine	13	-0.1705	0.2857	-0.1121	0.4462	0.002467	0.8262	0.843[0.530, 1.156]
Acne Vulgaris	isovalerylcarnitine	15	-0.2161	0.2869	0.0754	0.7325	0.031143	0.6185	0.806[0.408, 1.203]
Acne	2-methoxyacetamino	311	0.0046	0.2874	0.0095	0.1657	0.072241	0.0454	1.005[0.996,

Vulgaris	phen sulfate*								1.013]
Acne Vulgaris	2-hydroxystearate	29	-0.2035	0.2883	-0.4055	0.1448	0.185121	0.2095	0.816[0.440, 1.192]
Acne Vulgaris	X-11422--xanthine	6	-0.3075	0.2888	-0.4378	0.2423	0.945361	0.8541	0.735[0.167, 1.303]
Acne Vulgaris	phenyllactate (PLA)	15	0.1952	0.2981	0.2699	0.2413	0.252391	0.5911	1.216[0.848, 1.583]
Acne Vulgaris	pantothenate	22	-0.1752	0.2993	-0.0777	0.7078	0.068330	0.9803	0.839[0.508, 1.170]
Acne Vulgaris	glycerol 2-phosphate	29	-0.1252	0.3024	0.0103	0.9517	0.191962	0.4070	0.882[0.644, 1.120]
Acne	5-dodecenoate	15	0.1276	0.3122	0.1559	0.2689	0.113499	0.5411	1.136[0.889,

Vulgaris	(12:1n7)								1.384]
Acne Vulgaris	C-glycosyltryptophan*	14	0.3014	0.3162	0.1813	0.6446	0.975882	0.6474	1.352[0.762, 1.941]
Acne Vulgaris	X-13183--stearamide	6	0.1096	0.3164	0.1518	0.2786	0.588300	0.9335	1.116[0.901, 1.330]
Acne Vulgaris	1,5-anhydroglucitol (1,5-AG)	23	0.1556	0.3198	0.2534	0.2102	0.047000	0.7023	1.168[0.862, 1.475]
Acne Vulgaris	epiandrosterone sulfate	10	0.0620	0.3212	0.0509	0.4738	0.677286	0.4254	1.064[0.941, 1.186]
Acne Vulgaris	valerate	10	0.2004	0.3214	0.0159	0.9544	0.406886	0.6157	1.222[0.826, 1.618]
Acne	levulinate	46	-0.1739	0.3234	-0.3032	0.2018	0.116543	0.4842	0.840[0.495,

Vulgaris	(4-oxovalerate)								1.186]
Acne Vulgaris	HWESASXX*	4	-0.1530	0.3252	-0.1135	0.5337	0.314362	0.2658	0.858[0.553, 1.163]
Acne Vulgaris	methionine	22	-0.3261	0.3327	-0.4986	0.2974	0.814838	0.8003	0.722[0.062, 1.382]
Acne Vulgaris	myo-inositol	31	-0.1474	0.3402	-0.1808	0.4291	0.601884	0.7899	0.863[0.560, 1.166]
Acne Vulgaris	aspartylphenylalanine	4	-0.1437	0.3456	-0.0556	0.7546	0.398654	0.3044	0.866[0.567, 1.165]
Acne Vulgaris	biliverdin	13	0.1235	0.3513	0.0598	0.7342	0.268861	0.0706	1.131[0.872, 1.391]

Acne Vulgaris	1-palmitoleoylglycerophosphocholine*	10	0.1949	0.3585	0.2990	0.2937	0.716267	0.4039	1.215[0.799, 1.631]
Acne Vulgaris	2-hydroxyacetaminophen sulfate*	34	-0.0052	0.3616	-0.0036	0.6936	0.571137	0.5933	0.995[0.984, 1.006]
Acne Vulgaris	2-methylbutyroylcarnitine	18	0.1861	0.3629	-0.0356	0.8911	0.200820	0.2223	1.205[0.804, 1.605]
Acne Vulgaris	taurochenodeoxycholate	11	-0.0607	0.3696	-0.0225	0.7866	0.242380	0.1887	0.941[0.808, 1.074]
Acne Vulgaris	N-acetylalanine	21	0.3002	0.3728	0.4685	0.3118	0.621789	0.8704	1.350[0.690, 2.010]
Acne Vulgaris	hypoxanthine	20	0.1345	0.3732	0.0859	0.6913	0.414636	0.8562	1.144[0.848, 1.440]

Acne Vulgaris	xanthine	3	0.3300	0.3752	0.3172	0.4320	0.765902	0.6061	1.391[0.662, 2.120]
Acne Vulgaris	X-03056--N-[3-(2-Oxopyrrolidin-1-yl)propyl]acetamide	24	0.1262	0.3803	0.0804	0.6865	0.196269	0.6609	1.135[0.853, 1.416]
Acne Vulgaris	erythronate*	43	-0.1618	0.3822	0.0623	0.8035	0.150640	0.0793	0.851[0.488, 1.213]
Acne Vulgaris	creatine	6	-0.1470	0.3848	-0.3167	0.1411	0.301742	0.4566	0.863[0.532, 1.195]
Acne Vulgaris	margarate (17:0)	5	-0.2531	0.3875	-0.1805	0.6283	0.535482	0.9074	0.776[0.202, 1.351]
Acne Vulgaris	succinylcarnitine	37	0.1750	0.3902	0.1240	0.6273	0.016121	0.9689	1.191[0.792, 1.590]

Acne Vulgaris	paraxanthine	12	-0.0655	0.3974	-0.0009	0.9927	0.222427	0.1513	0.937[0.785, 1.088]
Acne Vulgaris	allantoin	17	-0.0817	0.4010	-0.1404	0.3013	0.571620	0.8169	0.922[0.731, 1.112]
Acne Vulgaris	undecanoate (11:0)	23	0.2067	0.4028	0.0639	0.8602	0.635227	0.6560	1.23[0.745, 1.714]
Acne Vulgaris	betaine	15	0.2267	0.4055	0.0377	0.8854	0.023716	0.2188	1.254[0.720, 1.788]
Acne Vulgaris	1-eicosadienoylglycerophosphocholine*	9	0.1107	0.4071	0.0031	0.9854	0.863219	0.1958	1.117[0.855, 1.379]
Acne Vulgaris	tetradecanedioate	14	0.0892	0.4138	0.1268	0.3575	0.137183	0.9106	1.093[0.879, 1.307]

Acne Vulgaris	cysteine-glutathione disulfide	8	0.0816	0.4138	0.0893	0.4988	0.837621	0.9053	1.085[0.889, 1.281]
Acne Vulgaris	pyroglutamine*	14	0.0824	0.4152	0.0369	0.7915	0.371138	0.8777	1.086[0.888, 1.284]
Acne Vulgaris	eicosapentaenoate (EPA; 20:5n3)	11	-0.1119	0.4256	-0.1473	0.4487	0.771264	0.9723	0.894[0.619, 1.169]
Acne Vulgaris	4-androsten-3beta,17 beta-diol disulfate 2*	14	-0.1268	0.4260	0.1154	0.5221	0.067314	0.2924	0.881[0.569, 1.193]
Acne Vulgaris	X-12990--docosapen taenoic acid (n6-DPA)	8	-0.1178	0.4265	-0.0771	0.6572	0.219525	0.1370	0.889[0.598, 1.179]
Acne Vulgaris	N-acetylglycine	12	-0.0831	0.4306	-0.0142	0.9139	0.288681	0.7828	0.920[0.714, 1.127]

Acne Vulgaris	DSGEGDFXAEGGGVR*	11	-0.0690	0.4351	-0.0885	0.4715	0.694420	0.3742	0.933[0.760, 1.107]
Acne Vulgaris	hippurate	14	-0.0645	0.4370	-0.1575	0.1658	0.951827	0.8460	0.938[0.775, 1.100]
Acne Vulgaris	X-11445--5-alpha-pregnan-3beta,20alpha-disulfate	14	0.0525	0.4440	0.0665	0.4300	0.152933	0.5116	1.054[0.920, 1.188]
Acne Vulgaris	alpha-ketoglutarate	15	-0.1185	0.4467	-0.2134	0.1865	0.004452	0.7363	0.888[0.583, 1.194]
Acne Vulgaris	phenylalanylphenylalanine	3	-0.1741	0.4496	-0.1333	0.5984	0.908358	0.9132	0.840[0.389, 1.292]
Acne Vulgaris	3-methylxanthine	13	-0.0646	0.4515	-0.0340	0.7636	0.984497	0.6293	0.937[0.769, 1.106]

Acne Vulgaris	X-13431--nonanoylcarnitine*	11	0.0926	0.4556	0.1502	0.3765	0.536953	0.8841	1.097[0.854, 1.340]
Acne Vulgaris	4-androsten-3beta,17beta-diol disulfate 1*	16	0.0391	0.4562	-0.0815	0.2359	0.378694	0.0573	1.040[0.937, 1.143]
Acne Vulgaris	oleoylcarnitine	7	0.1625	0.4574	0.2626	0.3586	0.400213	0.4663	1.176[0.748, 1.605]
Acne Vulgaris	uridine	17	0.2258	0.4615	0.1047	0.7922	0.896703	0.9061	1.253[0.652, 1.855]
Acne Vulgaris	gamma-glutamylphenylalanine	29	-0.1668	0.4617	-0.1173	0.6821	0.028635	0.5644	0.846[0.402, 1.291]
Acne Vulgaris	erythritol	24	0.0882	0.4632	0.2177	0.1615	0.131724	0.1331	1.092[0.857, 1.328]

Acne Vulgaris	cis-4-decenoyl carnitine	6	0.2436	0.4657	0.4040	0.1899	0.075459	0.1037	1.276[0.621, 1.930]
Acne Vulgaris	stachydrine	7	0.0488	0.4671	0.1039	0.1819	0.253200	0.9385	1.050[0.918, 1.182]
Acne Vulgaris	theobromine	4	-0.1394	0.4715	-0.0249	0.9108	0.294260	0.3060	0.870[0.490, 1.249]
Acne Vulgaris	gamma-glutamylvaline	12	-0.2422	0.4742	-0.0028	0.9939	0.019453	0.7153	0.785[0.121, 1.448]
Acne Vulgaris	gamma-glutamylthreonine*	10	-0.1180	0.4897	-0.1713	0.4337	0.796596	0.5405	0.889[0.554, 1.223]
Acne Vulgaris	cysteine	15	-0.1469	0.4898	-0.0096	0.9706	0.040939	0.8638	0.863[0.446, 1.280]

Acne Vulgaris	laurate (12:0)	35	0.1379	0.4944	0.0411	0.8624	0.010402	0.2533	1.148[0.752, 1.543]
Acne Vulgaris	1,6-anhydroglucose	14	0.0429	0.4968	0.0699	0.4098	0.349167	0.2843	1.044[0.920, 1.167]
Acne Vulgaris	indolelactate	12	0.1103	0.5001	0.0650	0.7784	0.561095	0.1915	1.117[0.796, 1.437]
Acne Vulgaris	heme*	13	0.0939	0.5008	0.0865	0.6021	0.080180	0.1808	1.098[0.825, 1.372]
Acne Vulgaris	4-hydroxyhippurate	7	0.0650	0.5074	0.0178	0.8888	0.836174	0.6659	1.067[0.875, 1.259]
Acne Vulgaris	1-linoleoylglycerophosphocholine	10	-0.1700	0.5106	-0.2217	0.4768	0.261957	0.8558	0.844[0.337, 1.350]

Acne Vulgaris	phenylalanine	3	-0.5598	0.5122	-0.1070	0.9168	0.470539	0.6370	0.571[-1.103, 2.246]
Acne Vulgaris	guanosine	13	-0.0470	0.5149	-0.0011	0.9907	0.963718	0.3867	0.954[0.813, 1.096]
Acne Vulgaris	cortisol	11	-0.0987	0.5203	-0.1264	0.5468	0.540010	0.8949	0.906[0.605, 1.207]
Acne Vulgaris	glycerate	14	0.1684	0.5237	0.1761	0.5925	0.196634	0.2759	1.183[0.666, 1.701]
Acne Vulgaris	palmitoylcarnitine	7	-0.1784	0.5264	-0.3255	0.2060	0.026038	0.7191	0.837[0.285, 1.389]
Acne Vulgaris	2-palmitoylglycerophosphocholine*	20	-0.0911	0.5324	0.1498	0.4667	0.363222	0.1241	0.913[0.627, 1.199]

Acne Vulgaris	histidine	5	-0.3377	0.5369	-0.5509	0.4300	0.757067	0.6260	0.713[-0.358, 1.785]
Acne Vulgaris	acetylcarnitine	17	-0.1283	0.5394	-0.0614	0.8062	0.005922	0.7680	0.880[0.470, 1.289]
Acne Vulgaris	hyodeoxycholate	12	-0.0371	0.5395	-0.0131	0.8721	0.622124	0.6388	0.964[0.845, 1.082]
Acne Vulgaris	inosine	8	0.0284	0.5428	0.0472	0.2932	0.062407	0.5340	1.029[0.937, 1.120]
Acne Vulgaris	stearate (18:0)	35	0.1470	0.5429	-0.0506	0.8590	0.004944	0.3241	1.158[0.685, 1.632]
Acne Vulgaris	estrone 3-sulfate	10	-0.0333	0.5430	-0.0322	0.6324	0.082669	0.5199	0.967[0.860, 1.074]

Acne Vulgaris	catechol sulfate	11	-0.0681	0.5464	-0.1377	0.3838	0.426070	0.1598	0.934[0.713, 1.156]
Acne Vulgaris	X-14450--phenylalanylleucine	5	-0.1022	0.5475	-0.3003	0.0601	0.077654	0.2451	0.903[0.570, 1.236]
Acne Vulgaris	2-hydroxybutyrate (AHB)	14	-0.1045	0.5498	0.0962	0.6735	0.280263	0.7227	0.901[0.558, 1.243]
Acne Vulgaris	5-oxoproline	17	0.1444	0.5575	0.3833	0.2131	0.263346	0.0454	1.155[0.673, 1.638]
Acne Vulgaris	gamma-glutamyltyrosine	34	-0.1204	0.5611	-0.1275	0.6032	0.040311	0.7792	0.887[0.480, 1.293]
Acne Vulgaris	4-acetaminophen sulfate	26	0.0044	0.5625	-0.0040	0.6558	0.037863	0.9071	1.004[0.990, 1.019]

Acne Vulgaris	gamma-glutamylisoleucine*	22	0.0840	0.5628	-0.0112	0.9555	0.259070	0.3975	1.088[0.803, 1.372]
Acne Vulgaris	leucine	169	0.1031	0.5668	0.3138	0.1938	0.055383	0.1179	1.109[0.756, 1.461]
Acne Vulgaris	isovalerate	4	0.1773	0.5701	0.2796	0.4476	0.467473	0.3317	1.194[0.582, 1.806]
Acne Vulgaris	phenol sulfate	13	-0.1059	0.5738	-0.1500	0.2831	0.000050	0.2650	0.900[0.530, 1.269]
Acne Vulgaris	serotonin (5HT)	14	0.0719	0.5812	0.0204	0.9101	0.863924	0.5363	1.075[0.819, 1.330]
Acne Vulgaris	tryptophan	151	-0.1031	0.5830	-0.0656	0.7900	0.044756	0.4774	0.902[0.534, 1.270]

Acne Vulgaris	1-myristoylglycerophosphocholine	4	-0.1657	0.5853	0.0347	0.8720	0.023861	0.3239	0.847[0.252, 1.443]
Acne Vulgaris	phenylacetate	6	0.0601	0.5988	0.0629	0.6611	0.872414	0.7587	1.062[0.838, 1.286]
Acne Vulgaris	saccharin	10	0.0247	0.6001	0.0384	0.5392	0.977179	0.8316	1.025[0.933, 1.117]
Acne Vulgaris	octanoylcarnitine	14	0.0700	0.6005	0.1160	0.3897	0.075878	0.6983	1.073[0.810, 1.335]
Acne Vulgaris	3-(4-hydroxyphenyl)lactate	18	0.1373	0.6040	0.1128	0.6696	0.000415	0.0582	1.147[0.628, 1.666]
Acne Vulgaris	1-arachidonoylglycerophosphoinositol*	14	0.0975	0.6055	0.1365	0.5947	0.944573	0.8383	1.102[0.733, 1.472]

Acne Vulgaris	N1-methyladenosine	8	-0.3194	0.6145	-0.3064	0.7020	0.282007	0.2002	0.727[-0.516, 1.970]
Acne Vulgaris	hexadecanedioate	16	-0.0644	0.6163	0.1139	0.4729	0.130709	0.4476	0.938[0.686, 1.189]
Acne Vulgaris	serine	25	-0.1077	0.6164	-0.1450	0.6402	0.438038	0.7802	0.898[0.476, 1.319]
Acne Vulgaris	choline	19	0.1632	0.6194	-0.0388	0.9322	0.572107	0.1510	1.177[0.533, 1.821]
Acne Vulgaris	docosahexaenoate (DHA; 22:6n3)	6	-0.0889	0.6218	-0.1470	0.5186	0.713694	0.6252	0.915[0.562, 1.268]
Acne Vulgaris	heptanoate (7:0)	29	0.1176	0.6220	-0.0157	0.9567	0.006388	0.8592	1.125[0.657, 1.592]

Acne Vulgaris	glycerol 3-phosphate (G3P)	14	-0.1014	0.6225	-0.1764	0.5370	0.984077	0.7397	0.904[0.500, 1.307]
Acne Vulgaris	stearidonate (18:4n3)	10	0.0619	0.6266	0.2004	0.2485	0.753532	0.2365	1.064[0.814, 1.313]
Acne Vulgaris	5alpha-androstan-3beta,17beta-diol disulfate	14	-0.0282	0.6309	0.0645	0.3894	0.328751	0.6055	0.972[0.857, 1.087]
Acne Vulgaris	X-12442--5,8-tetradecadienoate	11	0.0470	0.6326	0.0289	0.8327	0.547497	0.4815	1.048[0.855, 1.241]
Acne Vulgaris	glutaroyl carnitine	22	0.0695	0.6369	-0.0726	0.7028	0.160027	0.3352	1.072[0.783, 1.361]
Acne Vulgaris	1-linoleoylglycerol (1-monolinolein)	12	-0.0395	0.6436	0.0384	0.7420	0.459854	0.1862	0.961[0.794, 1.129]

Acne Vulgaris	n-Butyl Oleate	9	-0.1020	0.6516	0.1237	0.6457	0.241273	0.0545	0.903[0.460, 1.346]
Acne Vulgaris	dimethylarginine (SDMA + ADMA)	27	0.1211	0.6528	-0.1416	0.6398	0.012603	0.2484	1.129[0.601, 1.656]
Acne Vulgaris	bilirubin (E,E)*	8	0.0455	0.6533	0.0554	0.6663	0.798020	0.2227	1.047[0.848, 1.245]
Acne Vulgaris	lathosterol	11	0.0510	0.6561	0.0758	0.6484	0.367134	0.1397	1.052[0.828, 1.277]
Acne Vulgaris	phenylacetylglutamine	16	0.0478	0.6595	-0.1005	0.4335	0.076925	0.0340	1.049[0.836, 1.262]
Acne Vulgaris	glutamine	7	0.3405	0.6604	0.4393	0.4719	0.008655	0.7418	1.406[-0.113, 2.925]

Acne Vulgaris	X-11786--methylcysteine	11	-0.0459	0.6812	0.0362	0.8054	0.481977	0.6572	0.955[0.736, 1.174]
Acne Vulgaris	metoprolol acid metabolite*	25	0.0029	0.6813	0.0033	0.7339	0.841415	0.5446	1.003[0.989, 1.017]
Acne Vulgaris	beta-hydroxyisovalerate	16	0.0810	0.6825	0.0848	0.7308	0.062822	0.2509	1.084[0.696, 1.472]
Acne Vulgaris	isobutyrylcarnitine	9	0.0787	0.6833	0.0807	0.6759	0.050043	0.5299	1.082[0.704, 1.460]
Acne Vulgaris	gamma-glutamylleucine	27	0.0809	0.6841	0.0546	0.8531	0.745349	0.9844	1.084[0.695, 1.474]
Acne Vulgaris	cortisone	30	0.0740	0.6851	-0.2323	0.3737	0.797939	0.1780	1.077[0.719, 1.435]

Acne Vulgaris	glutamate	3	0.1971	0.6884	0.1471	0.7374	0.095006	0.2982	1.218[0.254, 2.181]
Acne Vulgaris	1,7-dimethylurate	10	-0.0375	0.6886	0.0062	0.9595	0.884600	0.9307	0.963[0.780, 1.147]
Acne Vulgaris	ursodeoxycholate	10	0.0285	0.6894	0.0673	0.4669	0.299326	0.2401	1.029[0.889, 1.169]
Acne Vulgaris	palmitoleate (16:1n7)	7	0.0796	0.6938	0.0853	0.7605	0.681958	0.2759	1.083[0.687, 1.479]
Acne Vulgaris	scyllo-inositol	8	0.0851	0.6958	0.0987	0.6925	0.125502	0.4457	1.089[0.662, 1.516]
Acne Vulgaris	4-acetamidobutanoate	26	-0.0748	0.6996	0.3253	0.2443	0.435024	0.3845	0.928[0.548, 1.308]

Acne Vulgaris	1-oleoylglycerol (1-monoolein)	15	0.0474	0.7018	-0.0026	0.9850	0.075168	0.8230	1.049[0.806, 1.291]
Acne Vulgaris	3-(cystein-S-yl)acetaminophen*	61	0.0031	0.7022	-0.0120	0.3076	0.639795	0.5776	1.003[0.987, 1.019]
Acne Vulgaris	X-11793--oxidized bilirubin*	11	-0.0790	0.7030	-0.0247	0.9126	0.034924	0.3271	0.924[0.518, 1.330]
Acne Vulgaris	adrenate (22:4n6)	9	0.0658	0.7066	0.0657	0.7615	0.297905	0.9172	1.068[0.725, 1.411]
Acne Vulgaris	2-stearoylglycerophosphocholine*	11	0.0525	0.7110	0.0502	0.8047	0.668147	0.8556	1.054[0.776, 1.332]
Acne Vulgaris	urate	18	0.0847	0.7117	-0.0266	0.9337	0.473757	0.8433	1.088[0.639, 1.537]

Acne Vulgaris	2-hydroxyglutarate	13	0.0509	0.7156	0.0658	0.7396	0.609545	0.5849	1.052[0.779, 1.326]
Acne Vulgaris	gamma-tocopherol	9	0.0338	0.7194	0.0140	0.9087	0.393701	0.5070	1.034[0.850, 1.219]
Acne Vulgaris	3-(3-hydroxyphenyl) propionate	10	0.0161	0.7265	-0.0083	0.8929	0.807850	0.4090	1.016[0.926, 1.107]
Acne Vulgaris	X-11593--O-methylascorbate*	37	0.0440	0.7354	-0.0506	0.7887	0.642602	0.7295	1.045[0.790, 1.300]
Acne Vulgaris	10-nonadecenoate (19:1n9)	7	0.0738	0.7356	-0.1056	0.6938	0.208628	0.1836	1.077[0.648, 1.505]
Acne Vulgaris	hydroquinone sulfate	15	-0.0171	0.7369	0.0264	0.6646	0.098574	0.0722	0.983[0.883, 1.083]

Acne Vulgaris	ornithine	11	-0.0871	0.7382	-0.2948	0.3807	0.275198	0.5478	0.917[0.406, 1.427]
Acne Vulgaris	glycodeoxycholate	4	0.0195	0.7409	0.0554	0.4414	0.553973	0.6150	1.020[0.904, 1.136]
Acne Vulgaris	oleate (18:1n9)	17	-0.0776	0.7444	-0.3571	0.2811	0.331469	0.4163	0.925[0.459, 1.392]
Acne Vulgaris	N2,N2-dimethylguanosine	43	0.0404	0.7451	-0.0411	0.8288	0.865606	0.4524	1.041[0.798, 1.285]
Acne Vulgaris	hydroxyisovaleroyl carnitine	7	0.0490	0.7597	0.1827	0.3998	0.542880	0.3659	1.050[0.736, 1.364]
Acne Vulgaris	gamma-glutamylmethionine*	8	0.0563	0.7632	0.2937	0.1179	0.082350	0.1599	1.058[0.692, 1.424]

Acne Vulgaris	nonadecanoate (19:0)	12	-0.0600	0.7744	0.0474	0.8585	0.280218	0.4272	0.942[0.532, 1.352]
Acne Vulgaris	pyridoxate	10	0.0379	0.7774	-0.1236	0.4160	0.168485	0.1591	1.039[0.776, 1.301]
Acne Vulgaris	taurocholate	12	0.0142	0.7788	0.0483	0.5142	0.563762	0.7182	1.014[0.916, 1.113]
Acne Vulgaris	4-methyl-2-oxopentanoate	11	0.0644	0.7889	0.0915	0.7687	0.893467	0.8728	1.067[0.595, 1.538]
Acne Vulgaris	isoleucine	14	0.0981	0.7908	0.4485	0.3781	0.657969	0.6685	1.103[0.378, 1.828]
Acne Vulgaris	ADpSGEGDFXAEGGGVR*	5	0.0353	0.7952	0.0464	0.7669	0.994905	0.7779	1.036[0.770, 1.302]

Acne Vulgaris	glycylvaline	7	0.0227	0.8093	0.0320	0.7984	0.473788	0.4967	1.023[0.839, 1.207]
Acne Vulgaris	1-linoleoylglycerophosphoethanolamine*	11	-0.0526	0.8095	0.0429	0.8689	0.147872	0.9230	0.949[0.521, 1.376]
Acne Vulgaris	X-04499--3,4-dihydroxybutyrate	15	0.0512	0.8123	0.0795	0.7946	0.597730	0.9124	1.053[0.630, 1.476]
Acne Vulgaris	lactate	9	-0.1011	0.8136	0.4642	0.2789	0.028542	0.1320	0.904[0.063, 1.744]
Acne Vulgaris	alpha-tocopherol	10	0.0396	0.8147	0.0204	0.9276	0.425818	0.5232	1.040[0.709, 1.372]
Acne Vulgaris	1-stearoylglycerophosphoinositol	5	0.0432	0.8151	-0.0572	0.7768	0.242412	0.1187	1.044[0.682, 1.406]

Acne Vulgaris	p-acetamidophenylglucuronide	40	0.0007	0.8167	0.0021	0.6185	0.731127	0.8347	1.001[0.995, 1.007]
Acne Vulgaris	dihomo-linolenate (20:3n3 or n6)	20	-0.0433	0.8170	-0.3982	0.0866	0.152541	0.0999	0.958[0.591, 1.324]
Acne Vulgaris	threitol	13	-0.0566	0.8216	-0.0242	0.8966	0.000001	0.9284	0.945[0.453, 1.437]
Acne Vulgaris	2-hydroxyisobutyrate	14	-0.0477	0.8253	0.0148	0.9548	0.190587	0.1452	0.953[0.530, 1.377]
Acne Vulgaris	eicosenoate (20:1n9 or 11)	9	0.0375	0.8263	-0.0683	0.7575	0.370486	0.2356	1.038[0.703, 1.373]
Acne Vulgaris	1-heptadecanoylglycerophosphocholine	7	0.0453	0.8356	0.0013	0.9961	0.285199	0.7739	1.046[0.618, 1.474]

Acne Vulgaris	erythrose	6	0.0377	0.8385	0.0955	0.6885	0.848439	0.3124	1.038[0.676, 1.401]
Acne Vulgaris	X-14189--leucylalanine	9	-0.0233	0.8395	0.0471	0.6990	0.079888	0.6340	0.977[0.751, 1.202]
Acne Vulgaris	1,3,7-trimethylurate	7	0.0117	0.8430	-0.0545	0.3491	0.098169	0.1808	1.012[0.896, 1.127]
Acne Vulgaris	3-methyl-2-oxovalerate	25	0.0462	0.8470	0.0596	0.8327	0.038453	0.6642	1.047[0.577, 1.517]
Acne Vulgaris	salicyluric glucuronide*	11	-0.0042	0.8519	-0.0075	0.8130	0.931818	0.9438	0.996[0.952, 1.040]
Acne Vulgaris	1-arachidonoylglycerophosphocholine*	15	0.0336	0.8533	0.0625	0.7867	0.917352	0.3310	1.034[0.678, 1.390]

Acne Vulgaris	glycerophosphorylcholine (GPC)	18	0.0261	0.8549	0.0120	0.9105	0.000056	0.8171	1.026[0.747, 1.306]
Acne Vulgaris	15-methylpalmitate (isobar with 2-methylpalmitate)	12	0.0332	0.8557	-0.1987	0.4392	0.577318	0.2448	1.034[0.676, 1.392]
Acne Vulgaris	glycerol	18	0.0269	0.8568	-0.0140	0.9492	0.467987	0.6546	1.027[0.735, 1.319]
Acne Vulgaris	1-stearoylglycerophosphocholine	11	-0.0300	0.8612	-0.1171	0.6212	0.511598	0.9579	0.970[0.634, 1.306]
Acne Vulgaris	2-linoleoylglycerophosphocholine*	12	0.0587	0.8622	-0.1245	0.6538	0.000323	0.5562	1.060[0.398, 1.723]
Acne Vulgaris	arginine	16	-0.0366	0.8642	0.0626	0.8265	0.341200	0.6376	0.964[0.545, 1.383]

Acne Vulgaris	X-14208--phenylalanylserine	9	0.0120	0.8684	0.0046	0.9615	0.811843	0.6195	1.012[0.870, 1.154]
Acne Vulgaris	aspartate	4	0.0332	0.8753	0.0780	0.7613	0.768608	0.7480	1.034[0.619, 1.449]
Acne Vulgaris	arabinose	4	0.0471	0.8779	-0.0239	0.9339	0.025662	0.6918	1.048[0.448, 1.649]
Acne Vulgaris	thymol sulfate	4	-0.0107	0.8793	-0.0053	0.9503	0.437404	0.3259	0.989[0.852, 1.127]
Acne Vulgaris	dodecanedioate	5	-0.0309	0.8839	0.1051	0.6807	0.288619	0.1372	0.970[0.555, 1.384]
Acne Vulgaris	valine	3	-0.1357	0.8913	-0.2533	0.8257	0.832224	0.6866	0.873[-1.073, 2.819]

Acne Vulgaris	1-oleoylglycerophosphocholine	13	-0.0287	0.8919	-0.0045	0.9877	0.705377	0.6105	0.972[0.557, 1.386]
Acne Vulgaris	taurodeoxycholate	11	-0.0079	0.8920	0.0135	0.8674	0.599675	0.2754	0.992[0.877, 1.107]
Acne Vulgaris	10-undecenoate (11:1n1)	21	-0.0121	0.8925	0.0665	0.5312	0.084277	0.3161	0.988[0.812, 1.164]
Acne Vulgaris	theophylline	12	0.0110	0.8955	-0.0110	0.9238	0.434541	0.0652	1.011[0.847, 1.176]
Acne Vulgaris	pyruvate	13	-0.0171	0.9042	0.0946	0.6528	0.516618	0.8679	0.983[0.705, 1.261]
Acne Vulgaris	ascorbate (Vitamin C)	13	0.0039	0.9043	-0.0294	0.5034	0.803291	0.0904	1.004[0.940, 1.068]

Acne Vulgaris	alpha-hydroxyisovalerate	13	0.0141	0.9105	0.0256	0.8775	0.285725	0.4128	1.014[0.768, 1.260]
Acne Vulgaris	X-12510--2-aminooctanoic acid	11	0.0104	0.9159	0.1011	0.2584	0.126742	0.2028	1.010[0.817, 1.204]
Acne Vulgaris	3-indoxyl sulfate	3	-0.0288	0.9175	-0.2715	0.2787	0.096933	0.6929	0.972[0.426, 1.517]
Acne Vulgaris	1-docosahexaenoylglycerophosphocholine*	6	0.0255	0.9185	-0.1843	0.5106	0.211236	0.0763	1.026[0.538, 1.514]
Acne Vulgaris	indolepropionate	15	-0.0144	0.9199	0.0921	0.5685	0.078830	0.7622	0.986[0.705, 1.266]
Acne Vulgaris	N-acetylthreonine	10	-0.0234	0.9212	0.2699	0.3898	0.288847	0.3738	0.977[0.514, 1.440]

Acne Vulgaris	1-palmitoylglycerophosphoethanolamine	22	0.0156	0.9252	0.0594	0.7646	0.081269	0.4177	1.016[0.691, 1.340]
Acne Vulgaris	X-14205--alpha-glutamyltyrosine	16	0.0070	0.9262	0.0272	0.7930	0.517652	0.6998	1.007[0.858, 1.156]
Acne Vulgaris	docosapentaenoate (n3 DPA; 22:5n3)	9	-0.0133	0.9276	0.0497	0.8115	0.440862	0.5664	0.987[0.699, 1.274]
Acne Vulgaris	glycochenodeoxycholate	12	0.0056	0.9282	0.0584	0.4725	0.330467	0.9946	1.006[0.884, 1.127]
Acne Vulgaris	linolenate [alpha or gamma; (18:3n3 or 6)]	4	-0.0233	0.9285	-0.1144	0.6998	0.318632	0.4074	0.977[0.467, 1.487]
Acne Vulgaris	quinate	5	-0.0078	0.9309	0.0035	0.9760	0.810989	0.4729	0.992[0.815, 1.170]

Acne Vulgaris	4-vinylphenol sulfate	9	-0.0067	0.9315	-0.0144	0.8810	0.378643	0.5263	0.993[0.840, 1.146]
Acne Vulgaris	X-12441--12-hydroxyeicosatetraenoate (12-HETE)	14	-0.0063	0.9341	0.0861	0.1963	0.000107	0.4920	0.994[0.843, 1.144]
Acne Vulgaris	caprylate (8:0)	35	-0.0126	0.9344	-0.1008	0.6354	0.042953	0.2870	0.987[0.688, 1.287]
Acne Vulgaris	benzoate	37	-0.0134	0.9392	-0.1726	0.5188	0.640435	0.4374	0.987[0.642, 1.331]
Acne Vulgaris	10-heptadecenoate (17:1n7)	4	0.0244	0.9411	0.0121	0.9724	0.211118	0.8398	1.025[0.378, 1.671]
Acne Vulgaris	tryptophan betaine	11	0.0036	0.9428	0.0396	0.5336	0.324511	0.5301	1.004[0.905, 1.102]

Acne Vulgaris	leucylleucine	10	0.0082	0.9493	-0.0043	0.9801	0.340931	0.6239	1.008[0.755, 1.261]
Acne Vulgaris	dehydroisoandrosterone sulfate (DHEA-S)	18	0.0052	0.9565	-0.1096	0.3967	0.472266	0.1969	1.005[0.820, 1.191]
Acne Vulgaris	palmitoyl sphingomyelin	39	0.0151	0.9569	0.5281	0.0431	0.000025	0.5053	1.015[0.468, 1.563]
Acne Vulgaris	cholate	8	-0.0031	0.9607	0.0068	0.9295	0.160959	0.9657	0.997[0.873, 1.121]
Acne Vulgaris	indoleacetate	18	-0.0054	0.9619	0.0047	0.9783	0.823305	0.7471	0.995[0.772, 1.217]
Acne Vulgaris	N-acetylornithine	914	-0.0007	0.9654	0.0191	0.5468	0.013464	0.4863	0.999[0.966, 1.032]

Acne Vulgaris	lysine	22	0.0123	0.9659	-0.0716	0.8659	0.557558	0.4431	1.012[0.448, 1.577]
Acne Vulgaris	2-aminobutyrate	38	0.0051	0.9761	0.1813	0.4640	0.592785	0.3226	1.005[0.672, 1.338]
Acne Vulgaris	3-methyl-2-oxobutyrate	13	-0.0089	0.9770	-0.0482	0.9030	0.267415	0.0624	0.991[0.387, 1.595]
Acne Vulgaris	glucose	28	-0.0077	0.9795	-0.0135	0.9709	0.030658	0.9241	0.992[0.403, 1.582]
Acne Vulgaris	palmitate (16:0)	23	-0.0065	0.9798	0.0689	0.8247	0.057108	0.3556	0.993[0.488, 1.499]
Acne Vulgaris	cotinine	14	0.0005	0.9830	0.0022	0.9378	0.883136	0.8470	1.000[0.959, 1.042]

Acne Vulgaris	glycine	18	0.0037	0.9833	-0.1628	0.5211	0.586231	0.7000	1.004[0.657, 1.351]
Acne Vulgaris	acetylphosphate	13	0.0084	0.9837	0.1385	0.7938	0.245136	0.9524	1.008[0.196, 1.820]
Acne Vulgaris	taurolithocholate 3-sulfate	9	0.0015	0.9877	-0.0046	0.9700	0.264671	0.2200	1.001[0.815, 1.188]
Acne Vulgaris	3-carboxy-4-methyl-5-propyl-2-furanpropanoate (CMPF)	10	-0.0005	0.9940	0.1009	0.2877	0.408209	0.6612	1.000[0.877, 1.122]
Acne Vulgaris	pro-hydroxy-pro	15	-0.0009	0.9965	0.0323	0.8952	0.095831	0.6060	0.999[0.581, 1.417]
Acne Vulgaris	myristoleate (14:1n5)	11	0.0005	0.9974	0.0303	0.8656	0.207517	0.9102	1.000[0.721, 1.280]
